# Supplementary material for: A fault-intrusion-tolerant system and deadline-aware algorithm for scheduling scientific workflow in the cloud
Source: PeerJ Comput Sci. 2021 Nov 2;7:e747. doi: 10.7717/peerj-cs.747 (PMC8576554; doi:10.7717/peerj-cs.747)
Supplement: Supplemental Information 6 [file peerj-cs-07-747-s006.zip › Myproject1.1/docs/org/cloudbus/cloudsim/class-use/Storage.html]

Uses of Interface org.cloudbus.cloudsim.Storage


JavaScript is disabled on your browser.


- Overview
- Package
- Class
- Use
- Tree
- Deprecated
- Index
- Help

- Prev
- Next

- Frames
- No Frames

- All Classes

## Uses of Interface org.cloudbus.cloudsim.Storage

- Packages that use Storage

  | Package | Description |
  | --- | --- |
  |  |  |
  | --- | --- |
  | org.cloudbus.cloudsim |  |
  | org.cloudbus.cloudsim.network.datacenter |  |
  | org.cloudbus.cloudsim.power |  |
  | org.workflowsim |  |
- - ### Uses of Storage in org.cloudbus.cloudsim

    Classes in org.cloudbus.cloudsim that implement Storage

    | Modifier and Type | Class and Description |
    | --- | --- |
    |  |  |
    | --- | --- |
    | `class` | `HarddriveStorage` An implementation of a storage system. |
    | `class` | `SanStorage` SANStorage represents a storage area network composed of a set of harddisks connected in a LAN. |

    Methods in org.cloudbus.cloudsim that return types with arguments of type Storage

    | Modifier and Type | Method and Description |
    | --- | --- |
    |  |  |
    | --- | --- |
    | `protected java.util.List<Storage>` | Datacenter.`getStorageList()` Gets the storage list. |

    Method parameters in org.cloudbus.cloudsim with type arguments of type Storage

    | Modifier and Type | Method and Description |
    | --- | --- |
    |  |  |
    | --- | --- |
    | `protected void` | Datacenter.`setStorageList(java.util.List<Storage> storageList)` Sets the storage list. |

    Constructor parameters in org.cloudbus.cloudsim with type arguments of type Storage

    Constructor and Description

    `Datacenter(java.lang.String name,
    DatacenterCharacteristics characteristics,
    VmAllocationPolicy vmAllocationPolicy,
    java.util.List<Storage> storageList,
    double schedulingInterval)`

    Allocates a new PowerDatacenter object.
  - ### Uses of Storage in org.cloudbus.cloudsim.network.datacenter

    Constructor parameters in org.cloudbus.cloudsim.network.datacenter with type arguments of type Storage

    Constructor and Description

    `NetworkDatacenter(java.lang.String name,
    DatacenterCharacteristics characteristics,
    VmAllocationPolicy vmAllocationPolicy,
    java.util.List<Storage> storageList,
    double schedulingInterval)`

    Allocates a new NetworkDatacenter object.
  - ### Uses of Storage in org.cloudbus.cloudsim.power

    Constructor parameters in org.cloudbus.cloudsim.power with type arguments of type Storage

    Constructor and Description

    `PowerDatacenter(java.lang.String name,
    DatacenterCharacteristics characteristics,
    VmAllocationPolicy vmAllocationPolicy,
    java.util.List<Storage> storageList,
    double schedulingInterval)`

    Instantiates a new datacenter.

    `PowerDatacenterNonPowerAware(java.lang.String name,
    DatacenterCharacteristics characteristics,
    VmAllocationPolicy vmAllocationPolicy,
    java.util.List<Storage> storageList,
    double schedulingInterval)`

    Instantiates a new datacenter.
  - ### Uses of Storage in org.workflowsim

    Classes in org.workflowsim that implement Storage

    | Modifier and Type | Class and Description |
    | --- | --- |
    |  |  |
    | --- | --- |
    | `class` | `ClusterStorage` ClusterStorage is an extention of HarddriveStorage and it is used as a local storage system of a vm |

    Constructor parameters in org.workflowsim with type arguments of type Storage

    Constructor and Description

    `DatacenterExtended(java.lang.String name,
    DatacenterCharacteristics characteristics,
    VmAllocationPolicy vmAllocationPolicy,
    java.util.List<Storage> storageList,
    double schedulingInterval)`

- Overview
- Package
- Class
- Use
- Tree
- Deprecated
- Index
- Help

- Prev
- Next

- Frames
- No Frames

- All Classes
